# Gender differences in neurocognitive impairment among first-episode, drug-naïve schizophrenia patients: a cross-sectional study

**DOI:** 10.3389/fpsyt.2025.1642291

**Published:** 2025-08-04

**Authors:** Qing Zhang, AiMin Sui, Min Su, MingLiang Ju, YanYan Wei, XiaoChen Tang, LiHua Xu, HuiRu Cui, YingYing Tang, ZhengHui Yi, HaiChun Liu, Jin Gao, JiJun Wang, TianHong Zhang

**Affiliations:** ^1^ Shanghai Mental Health Center, Shanghai Jiaotong University School of Medicine, Shanghai Engineering Research Center of Intelligent Psychological Evaluation and Intervention, Shanghai Key Laboratory of Psychotic Disorders, Shanghai, China; ^2^ Department of Psychiatry, Taizhou Second People’s Hospital, Zhejiang, China; ^3^ Department of Psychiatry, Ningde Rehabilitation Hospital, Fujian, China; ^4^ Department of Automation, Shanghai Jiao Tong University, Shanghai, China; ^5^ Department of Clinical Psychology, Qilu Hospital (Qingdao), Cheeloo College of Medicine, Shandong University, Qingdao, Shandong, China; ^6^ Department of Psychiatry, Nantong Fourth People’s Hospital and Nantong Brain Hospital, Suzhou, China

**Keywords:** psychosis, cognition, gender, first-episode, schizophrenia

## Abstract

**Introduction:**

Cognitive impairment is a core feature of first-episode schizophrenia (FES), yet the influence of biological sex on its manifestation remains under characterized. Existing evidence suggests sex differences in cognitive profiles among chronic schizophrenia patients, but whether these patterns emerge in drug-naïve FES patients—and how they relate to clinical symptoms—requires clarification.

**Methods:**

We recruited 382 drug-naïve FES patients and 522 healthy controls (HCs) matched for age and education. Cognitive function was assessed using the MATRICS Consensus Cognitive Battery (MCCB). Clinical symptoms were evaluated via the Positive and Negative Syndrome Scale (PANSS).

**Results:**

FES patients demonstrated global cognitive deficits compared to HCs. When stratified by diagnostic group (FES and HC), males outperformed females in planning and problem-solving, as measured by the Neuropsychological Assessment Battery Mazes subtest (*p* < 0.001), whereas females showed superior performance in visuospatial memory, as assessed by the Brief Visuospatial Memory Test–Revised (*p* < 0.001) in both groups. When stratified by gender, FES patients exhibited similar patterns of impairment severity relative to their gender-matched HCs: both male and female FES patients showed the most pronounced deficits in processing speed (BACS) and sustained attention (CPT), with effect sizes of 1.64 and 1.52 for males, and 1.36 and 1.48 for females, respectively. Correlational analyses revealed that male FES patients’ cognitive impairments were specifically associated with negative symptoms, while female impairments correlated broadly with all PANSS domains. Correlational analyses revealed that in FEP patients, male cognitive impairments were specifically associated with negative symptoms, while female impairments showed broad associations with all domains of the PANSS.

**Conclusion:**

FES manifests as sex-divergent cognitive profiles, with males showing executive/processing speed deficits tied to negative symptoms and females exhibiting memory impairments with broader symptom associations. These findings underscore the need for sex-sensitive approaches in characterizing cognitive dysfunction in early psychosis.

## 1 Introduction

Cognitive impairment has emerged as a critical factor in the developmental trajectory of psychosis (1, 2), with research indicating that subtle yet measurable cognitive deficits often precede the onset of full-blown psychotic symptoms during the clinical high-risk state (3, 4). These pre-psychotic cognitive impairments, though less severe than those observed in first-episode schizophrenia (FES) patients, involve domains such as working memory (5, 6), processing speed (7, 8), and social cognition (9, 10), which are known to deteriorate further as the disorder progresses (11). Notably, cognitive dysfunction has been linked to the duration of untreated psychosis (12, 13) and the duration of untreated prodromal psychosis (14), suggesting a potential role in modulating the transition from subclinical to clinical psychosis (15–18). Notably, the loss of insight-a hallmark of psychotic episodes-is profoundly shaped by cognitive function (19), as intact cognitive processes are essential for self-awareness of illness and symptom appraisal.

Previous studies have identified sex differences in cognitive impairment among patients with schizophrenia. Clinical evidence indicates that sex differences in cognitive impairment among schizophrenia patients may underlie divergent symptom profiles and treatment responses. For example, a study on chronic schizophrenia patients revealed that female patients exhibited more severe deficits in attention and reasoning/problem-solving, with attention independently associated with negative symptoms, whereas male patients showed stronger links between negative symptoms and verbal learning/memory as well as social cognition (20). However, these findings are constrained by critical limitations: most investigations included medicated patients with long disease durations, leaving the specific impact of psychosis itself on cognitive sex differences unclear. A pivotal gap exists in understanding whether these gender-related cognitive disparities emerge at the onset of psychosis or develop as a consequence of disease progression and medication exposure.

These limitations are further compounded by the fact that the majority of prior research has focused exclusively on chronic-phase patients (21–23), in whom cognitive deficits are confounded by factors like medication use, disease progression, and comorbidities. As a result, it remains challenging to disentangle the specific impact of psychosis on cognitive function across genders. Understanding sex differences in cognitive impairment during the early stages of psychosis, particularly in FES patients, is of paramount importance. Early identification of these differences could provide critical insights into the underlying psychological and neurobiological mechanisms unique to each gender, which may guide the development of more targeted and effective interventions. For example, such mechanisms may involve sex-specific hormonal influences on hippocampal function (24), cortical thickness disparities in visuospatial processing regions (25), or neurotransmitter system variations (e.g., dopamine sensitivity in the striatum) (26), all of which are supported by preclinical and clinical evidence linking biological sex to cognitive profiles in psychosis. Moreover, characterizing cognitive profiles at the onset of schizophrenia can help clinicians better predict disease trajectories and tailor personalized treatment strategies, ultimately improving outcomes and quality of life for patients.

Given the critical knowledge gaps in understanding sex-specific cognitive impairments during the first episode of psychosis, the present study aims to address this void. Our hypothesis posits that FES patients will exhibit distinct gender-divergent cognitive profiles compared to healthy controls, and that these differences may be associated with varying patterns of clinical symptom expression. Specifically, we propose that biological sex influences the nature and severity of cognitive deficits in early psychosis. Given that sex-specific cognitive profiles may underpin differential treatment responses and disease trajectories, characterizing these differences in drug-naïve FES patients could facilitate the development of gender-tailored diagnostic tools and precision interventions for early psychosis. Furthermore, we hypothesize that gender-specific cognitive impairments in FES patients will demonstrate differential associations with psychotic symptoms, highlighting the need for sex-sensitive approaches to characterize cognitive dysfunction in early psychosis. To test these hypotheses, we compared cognitive performance and symptom correlations between drug-naïve FES patients and healthy controls, with a focus on disentangling sex effects.

## 2 Methods

### 2.1 Participants

A total of 382 FES patients and 522 healthy controls (HC) were recruited for this study. The FES participants, aged 18–45 years, met the Diagnostic and Statistical Manual of Mental Disorders, Fourth Edition, Text Revision (DSM-IV-TR) criteria for psychotic disorders and were recruited from the Shanghai Mental Health Center (SMHC). Data collection occurred between 2016 and 2021 as part of the National Key R&D Program of China. All FEP patients completed the Chinese versions of the Positive and Negative Symptom Scale (PANSS) (27) and the Measurement and Treatment Research to Improve Cognition in Schizophrenia (MATRICS) Consensus Cognitive Battery (MCCB) (28–30). These patients had no prior exposure to psychotropic medications and were excluded if they had a history of substance abuse or dependence.

The HC group, recruited from the community, consisted of individuals without a personal history of mental illness or a family history of mental disorders among first-degree relatives. Their ages were carefully matched to those of the FES group to ensure comparability. The study was conducted at SMHC, with all procedures approved by the SMHC Research Ethics Committee (IRB2016-009). Written informed consent was obtained from each participant, and all methods adhered to the ethical guidelines of relevant national and institutional review boards for human research, in accordance with the 1975 Declaration of Helsinki (2008 revision).

### 2.2 Clinical psychopathology assessments

The PANSS was employed to evaluate clinical psychopathology. Comprising 30 items, the PANSS is categorized into three distinct subscales: the positive symptoms subscale (PANSS-P; items P1–7), the negative symptoms subscale (PANSS-N; N1–7), and the general psychopathology subscale (PANSS-G; G1–16). Each item was rated on a 7-point Likert scale, where 1 indicated the absence of a symptom and 7 signified an extreme manifestation.

Five senior psychiatrists, all of whom had completed specialized training for this research, conducted structured clinical interviews. Rigorous training ensured high inter-rater reliability, with coefficients ranging from 0.76 to 0.92 across the trained assessors. To facilitate meaningful comparisons among the three PANSS subscales, a Z-score transformation was applied to the entire sample, standardizing scores and enabling uniform interpretation of psychopathological severity across subscales.

### 2.3 Neurocognitive functions assessments

Neurocognitive function was systematically evaluated using the Chinese version of the MCCB, with all assessments strictly following the standardized protocols detailed in the test manual. The assessment battery encompassed nine subtests, each designed to target specific cognitive domains: the Trail Making Test (TMT) gauged visual attention and task - switching capabilities; BACS symbol coding from the Brief Assessment of Cognition in Schizophrenia assessed processing speed; the Revised Hopkins Verbal Learning Test (HVLT) evaluated verbal learning and memory; the Wechsler Memory Scale (WMS) examined overall memory functions; NAB mazes from the Neuropsychological Assessment Battery tested planning and problem - solving skills; the Revised Brief Visuospatial Memory Test (BVMT) focused on visuospatial memory; the Category Fluency test (CF) measured verbal fluency; the Mayer - Salovey - Caruso Emotional Intelligence Test (MSCEIT) assessed emotional intelligence; and the Continuous Performance Test - Identical Pairs (CPT) evaluated sustained attention. Notably, for all subtests except TMT Part A, higher scores signified better cognitive performance, whereas lower scores in TMT Part A indicated superior performance. The assessments were conducted by five trained raters, and the inter - rater reliability of the MCCB across these raters was robust, with coefficients ranging from 0.80 to 0.96.

### 2.4 Statistical analyses

All statistical analyses were conducted using appropriate software. Demographic characteristics between groups were compared with independent-samples t-tests for continuous variables and chi-square tests for categorical variables to check for baseline differences. To assess group differences in cognitive performance, effect sizes (calculated as the difference in means divided by the pooled standard deviation) were computed, and independent-samples t-tests were performed, with statistical significance set at *p* < 0.05. For exploring the relationships between cognitive test scores and PANSS clinical symptoms, linear regression models were employed. F-values were used to determine the significance of the regression slopes, and a p-value threshold of less than 0.05 indicated significant associations. To ensure comparability across various cognitive tests and scales, all data underwent Z-score transformation prior to analysis.

## 3 Results

### 3.1 Demographic, clinical and cognitive characteristics

The **Table 1** presents the socio-demographic, clinical, and cognitive characteristics of 522 HC and 382 patients with FES. There was no significant difference in age between the two groups (*t* = 1.007, *p* = 0.314), but a significant disparity in sex distribution (*χ²* = 9.688, *p* = 0.002), with a higher proportion of males in the FES group. Education levels were comparable (*t* = 0.385, *p* = 0.700), yet FES patients’ parents had significantly lower education levels (father: *t* = 3.798, *p* < 0.001; mother: *t* = 4.510, *p* < 0.001). FES patients exhibited positive (mean = 21.62, SD = 5.933), negative (mean = 17.55, SD = 7.230), and general symptoms (mean = 39.37, SD = 7.942), with a total PANSS score of 78.53 (SD = 16.194). Notably, FES patients showed significantly poorer performance in all MCCB cognitive domains, including TMT, BACS, HVLT, WMS, NAB, BVMT, CF, MSCEIT, and CPT (all *p* < 0.001).

**Table 1:** Socio-demographic, clinical, cognitive characteristics for healthy controls (HC) and patients with first episode schizophrenia (FES).

Variables		HC		FES		*χ^2^*/*t*	*p*
		N/Means	%/SD.	N/Means	%/SD.		
Cases		522		382		-	-
Age		26.19	4.280	25.87	5.216	1.007	0.314
Sex	Male	235	45.0%	212	55.5%	9.688	0.002
	Female	287	55.0%	170	44.5%		
Education		14.99	2.601	14.92	3.077	0.385	0.700
Father-Education		11.09	3.466	10.01	3.680	3.798	<0.001
Mather-Education		10.26	3.758	8.86	3.980	4.510	<0.001
PANSS scores							
Positive symptoms		-	-	21.62	5.933	-	-
Negative symptoms		-	-	17.55	7.230	-	-
General symptoms		-	-	39.37	7.942	-	-
Total score		-	-	78.53	16.194	-	-
MCCB scores							
TMT		27.81	9.873	45.50	23.957	-15.206	<0.001
BACS		65.10	10.000	48.05	11.678	23.576	<0.001
HVLT		26.87	4.272	21.22	5.646	17.114	<0.001
WMS		16.59	2.886	14.75	3.365	8.811	<0.001
NAB		19.57	4.865	13.08	6.717	16.872	<0.001
BVMT		28.61	5.055	21.47	7.749	16.762	<0.001
CF		24.13	5.647	18.80	5.441	14.236	<0.001
MSCEIT		87.16	8.441	80.66	10.323	10.390	<0.001
CPT		3.0002	.60826	1.9920	.79106	21.658	<0.001

*t* values are from independent t-tests, and *χ* ^²^ values are from the kappa test. MCCB stands for the Measurement and Treatment Research to Improve Cognition in Schizophrenia, a comprehensive battery of cognitive tests. TMT refers to Trail Making Test, assessing visual attention and task-switching; BACS symbol coding from the Brief Assessment of Cognition in Schizophrenia, evaluating processing speed; HVLT is the Revised Hopkins Verbal Learning Test, evaluating verbal learning and memory; WMS represents the Wechsler Memory Scale; NAB mazes from the Neuropsychological Assessment Battery, testing planning and problem-solving; BVMT is the Revised Brief Visuospatial Memory Test, focusing on visuospatial memory; CF is the Category Fluency test, evaluating verbal fluency; MSCEIT is the Mayer-Salovey-Caruso Emotional Intelligence Test, measuring emotional intelligence; CPT is the Continuous Performance Test-Identical Pairs, assessing sustained attention. For all tests except TMT, higher scores indicate better cognitive functioning, while lower scores in TMT suggest superior cognitive performance. PANSS stands for the Positive and Negative Syndrome Scale.

In addition to cognitive assessments, we compared PANSS scores between male and female FES patients (**Table 2**). No significant gender differences were observed in positive symptoms (male: 21.17 ± 6.36 *vs*. female: 22.18 ± 5.32, *t* = −1.65, *p* = 0.099), negative symptoms (17.52 ± 7.43 *vs*. 17.59 ± 7.00, *t* = −0.09, *p* = 0.926), general psychopathology (39.48 ± 7.72 *vs*. 39.22 ± 8.23, *t* = 0.32, *p* = 0.753), or total PANSS scores (78.17 ± 16.16 *vs*. 78.99 ± 16.27, *t* = −0.49, *p* = 0.624). Although male patients trended toward lower positive symptom scores (*p* = 0.099), this difference did not reach statistical significance.

**Table 2:** Gender comparison of Positive and Negative Syndrome Scale (PANSS) scores in first-episode schizophrenia (FES) patients.

Variables	Male		Female		*t*	*p*
	N/Means	%/SD.	N/Means	%/SD.		
Cases	212		170		-	-
Positive symptoms	21.17	6.362	22.18	5.315	-1.652	.099
Negative symptoms	17.52	7.426	17.59	7.000	-.093	.926
General symptoms	39.48	7.720	39.22	8.232	.315	.753
Total score	78.17	16.163	78.99	16.269	-.490	.624

*t* values are from independent t-tests. PANSS stands for the Positive and Negative Syndrome Scale, which is a widely used rating scale for assessing the severity of positive symptoms, negative symptoms, and general psychopathological symptoms in patients with schizophrenia.

### 3.2 HC-FES stratified cognitive differences between males and females

**Figure 1:**
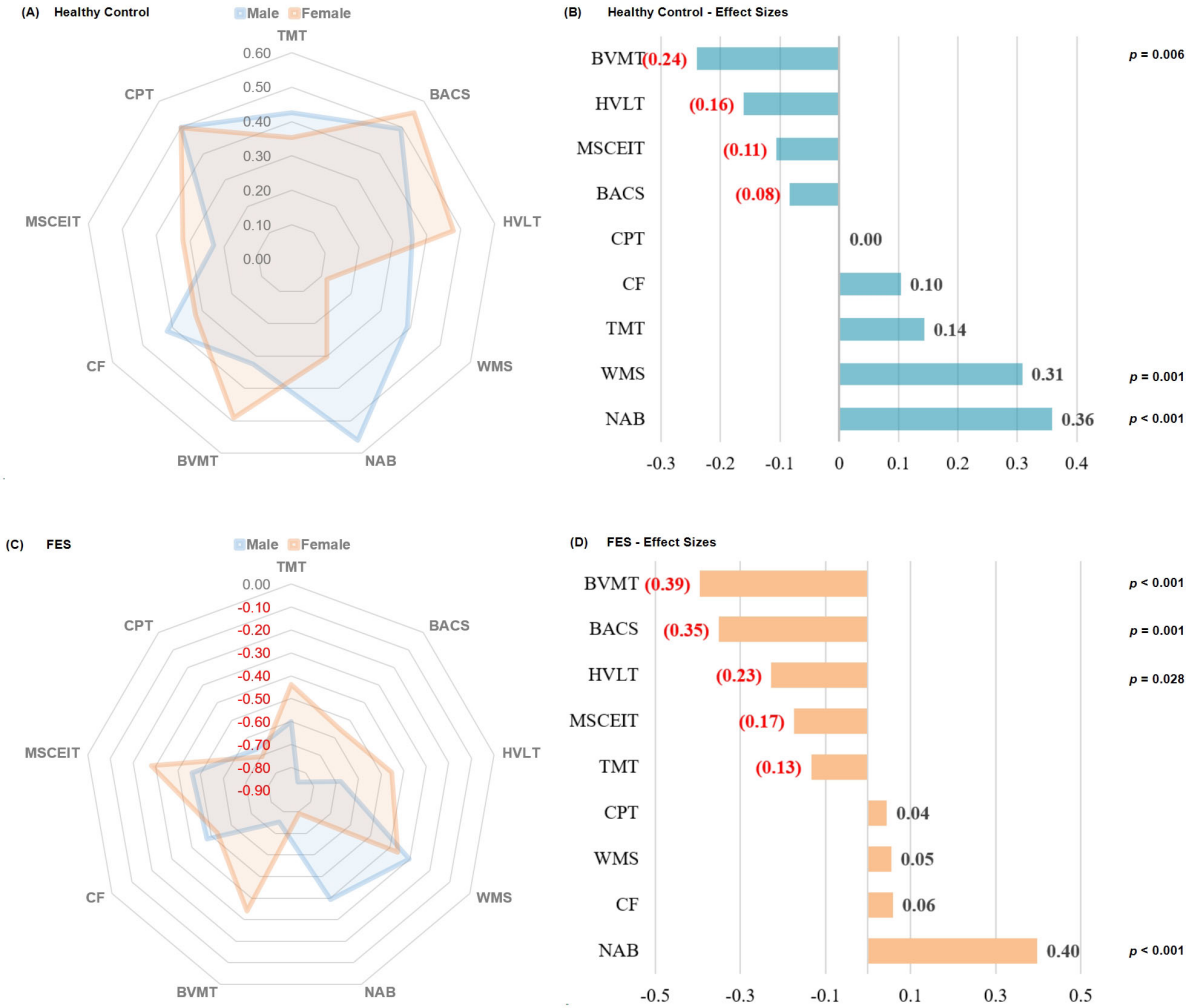
Radar plots and bar charts of effect sizes illustrating gender-based cognitive differences in healthy controls (HC) and first - episode schizophrenia (FES) patients. The radar plots display male and female performance across various cognitive tests, while the bar charts quantify the magnitude of gender differences with corresponding statistical significance levels. TMT refers to Trail Making Test, assessing visual attention and task-switching; BACS symbol coding from the Brief Assessment of Cognition in Schizophrenia, evaluating processing speed; HVLT is the Revised Hopkins Verbal Learning Test, evaluating verbal learning and memory; WMS represents the Wechsler Memory Scale; NAB mazes from the Neuropsychological Assessment Battery, testing planning and problem-solving; BVMT is the Revised Brief Visuospatial Memory Test, focusing on visuospatial memory; CF is the Category Fluency test, evaluating verbal fluency; MSCEIT is the Mayer-Salovey-Caruso Emotional Intelligence Test, measuring emotional intelligence; CPT is the Continuous Performance Test-Identical Pairs, assessing sustained attention. For the purpose of enhancing comparability, all samples underwent Z-score transformation. Since for TMT, a lower score indicates better performance, after the Z-score transformation, a negative sign was added to make it consistent with other tests. The calculation method for Effect Size is calculated by subtracting the mean of the second group from the mean of the first group. The resulting difference is then divided by the square root of the average of the squared standard deviations of the two groups.

The **Figure 1** was utilized to examine gender-related cognitive disparities within diagnostic groups, we compared cognitive performance between males and females in both HCs and FES patients.

In HCs, significant gender differences were observed across multiple cognitive domains. Males demonstrated superior performance in NAB and WMS, while females outperformed males in BVMT.

In FES patients, gender-specific cognitive patterns were also evident. Males showed better performance in NAB (effect size = 0.40, *p* < 0.001), while females exhibited superiority in BVMT, BACS and HVLT.

### 3.3 Gender-stratified cognitive differences between HC and FES patients

**Figure 2:**
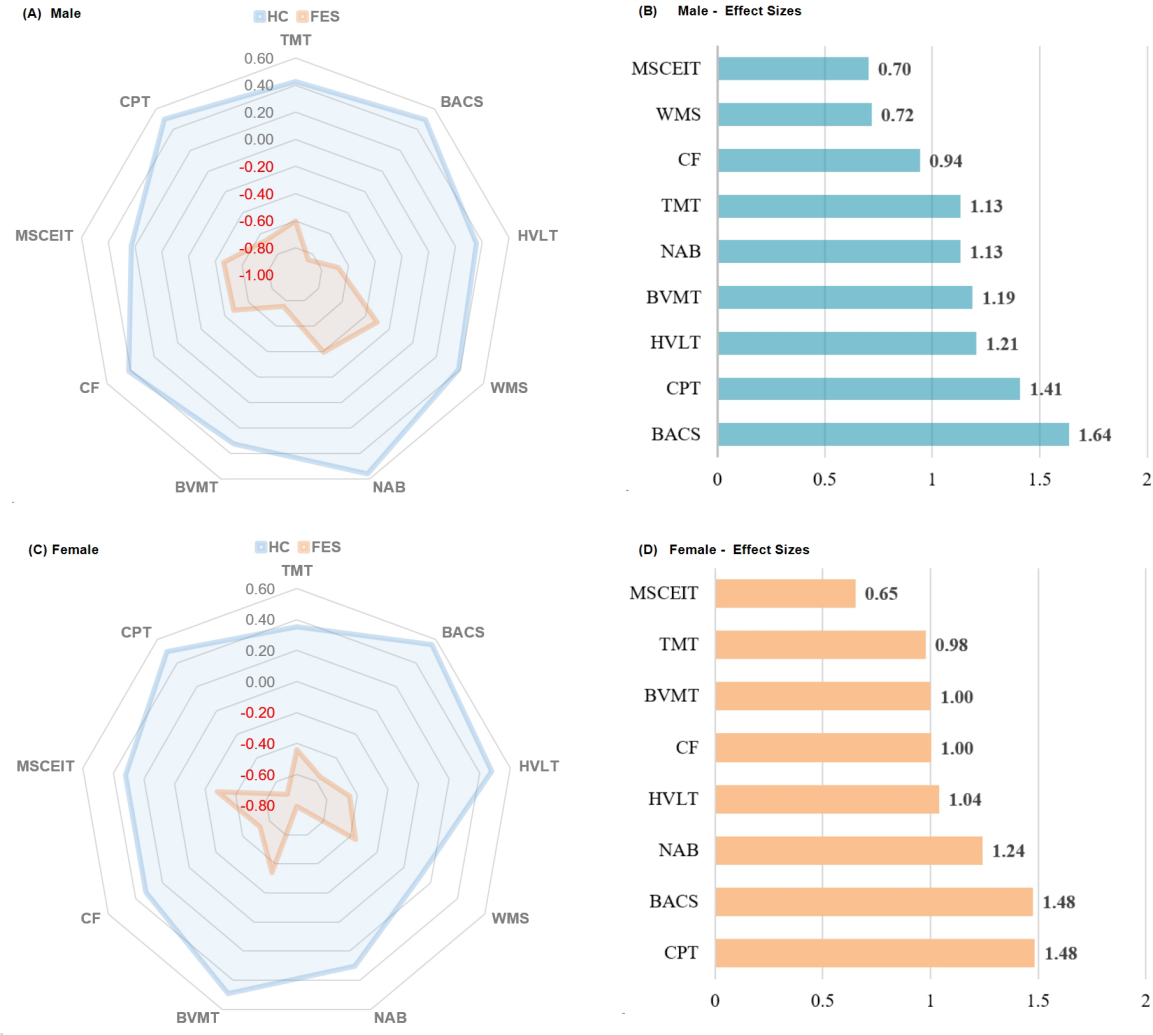
Radar plots and bar charts of effect sizes showing cognitive differences between healthy controls (HC) and first - episode schizophrenia (FES) patients, stratified by gender. The radar plots display the cognitive performance of HC and FES in different tests for males and females respectively, while the bar charts quantify the magnitude of differences between HC and FES with corresponding effect sizes. TMT refers to Trail Making Test, assessing visual attention and task-switching; BACS symbol coding from the Brief Assessment of Cognition in Schizophrenia, evaluating processing speed; HVLT is the Revised Hopkins Verbal Learning Test, evaluating verbal learning and memory; WMS represents the Wechsler Memory Scale; NAB mazes from the Neuropsychological Assessment Battery, testing planning and problem-solving; BVMT is the Revised Brief Visuospatial Memory Test, focusing on visuospatial memory; CF is the Category Fluency test, evaluating verbal fluency; MSCEIT is the Mayer-Salovey-Caruso Emotional Intelligence Test, measuring emotional intelligence; CPT is the Continuous Performance Test-Identical Pairs, assessing sustained attention. For the purpose of enhancing comparability, all samples underwent Z-score transformation. Since for TMT, a lower score indicates better performance, after the Z-score transformation, a negative sign was added to make it consistent with other tests. The calculation method for Effect Size is calculated by subtracting the mean of the second group from the mean of the first group. The resulting difference is then divided by the square root of the average of the squared standard deviations of the two groups.

**Figure 2** was employed to characterize the severity of cognitive deficits in FES relative to HCs, stratified by gender, we compared performance across all cognitive domains for males and females separately.

In males, FES patients exhibited significant impairments relative to male HCs across all cognitive domains, with the largest effect sizes observed in BACS, CPT, HVLT, and BVMT. Smaller but still significant deficits were noted in NAB, TMT, CF, WMS, and MSCEIT.

In females, FES patients also demonstrated significant impairments relative to female HCs across all cognitive domains. The largest effect sizes were observed in CPT, BACS, NAB, and HVLT. Smaller significant deficits were noted in CP, BVMT, TMT, and MSCEIT.

### 3.4 Gender-stratified correlations

**Figure 3:**
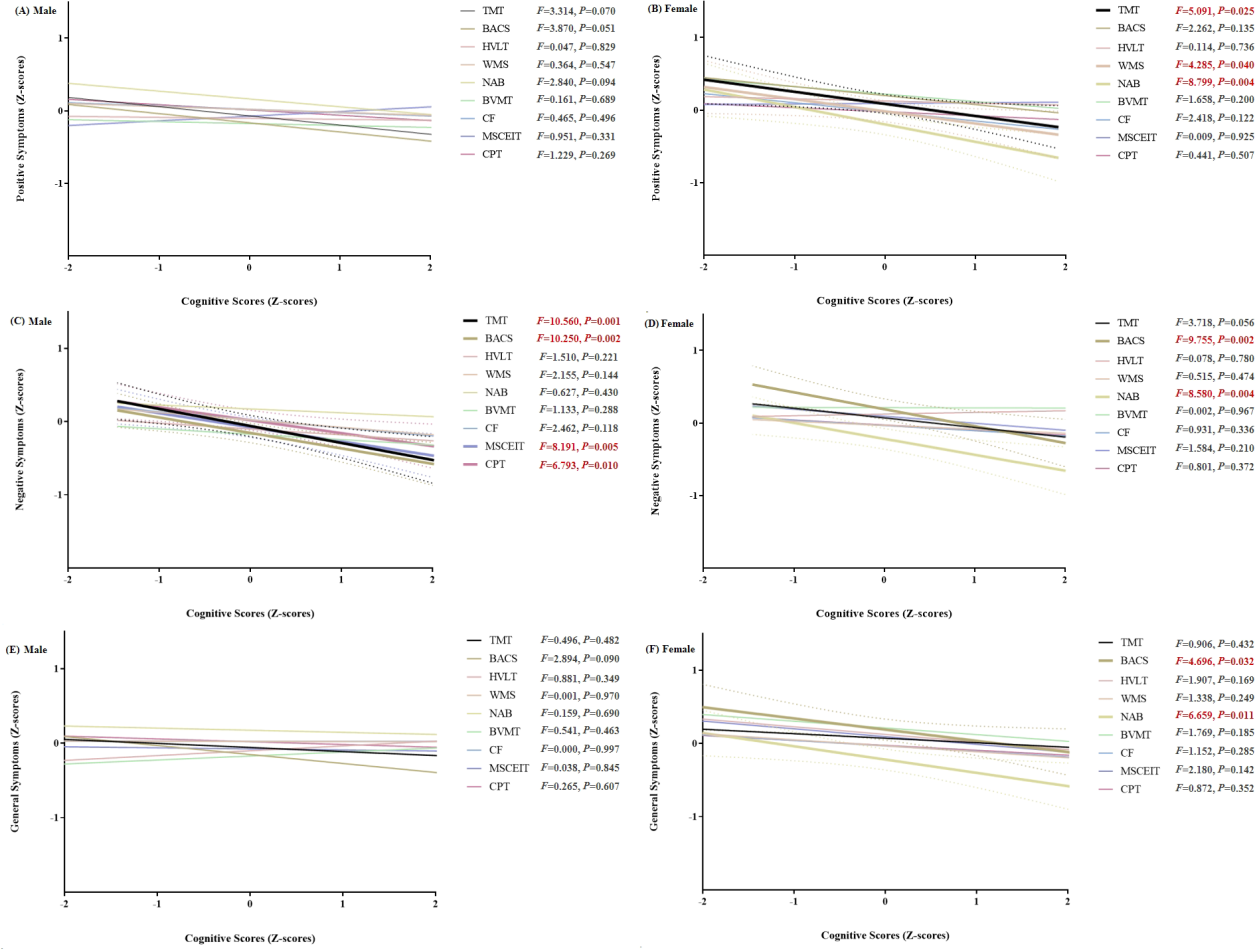
Plots illustrating the correlations between cognitive test scores and PANSS clinical symptoms, stratified by gender, in FES patients. TMT refers to Trail Making Test, assessing visual attention and task-switching; BACS symbol coding from the Brief Assessment of Cognition in Schizophrenia, evaluating processing speed; HVLT is the Revised Hopkins Verbal Learning Test, evaluating verbal learning and memory; WMS represents the Wechsler Memory Scale; NAB mazes from the Neuropsychological Assessment Battery, testing planning and problem-solving; BVMT is the Revised Brief Visuospatial Memory Test, focusing on visuospatial memory; CF is the Category Fluency test, evaluating verbal fluency; MSCEIT is the Mayer-Salovey-Caruso Emotional Intelligence Test, measuring emotional intelligence; CPT is the Continuous Performance Test-Identical Pairs, assessing sustained attention. For the purpose of enhancing comparability, FES samples underwent Z-score transformation. Since for TMT, a lower score indicates better performance, after the Z-score transformation, a negative sign was added to make it consistent with other tests. The F-value is used to determine whether the slope is significant, and a p-value less than 0.05 indicates significance.

**Figure 3** was used to explore the correlations between cognitive test scores and PANSS clinical symptoms, stratified by gender, among FES patients. In male FES patients, as presented in sub-figures (a), (c), and (e), there was no significant correlation between positive symptoms and cognitive tests. For negative symptoms, significant correlations were found with TMT, BACS, MSCEIT, and CPT cognitive tests (*p* < 0.05). There was no significant correlation between general psychopathological symptoms and cognitive tests. In female FES patients, shown in sub - figures (b), (d), and (f), the correlation patterns were distinct from those of males. For positive symptoms, significant correlations were found with TMT, WMS, and NAB cognitive tests (*p* < 0.05). For negative symptoms, significant correlations were found with BACS, and NAB cognitive tests (*p* < 0.05). For general psychopathological symptoms, significant correlations were found with BACS, and NAB cognitive tests (*p* < 0.05).

## 4 Discussion

The study’s strengths include a relatively large sample size, comprehensive cognitive assessment via the MCCB, inclusion of medication-naïve FES patients to exclude drug effects, and exclusion of substance abuse cases to rule out psychoactive-induced impairments. Key findings reveal gender-stratified cognitive differences: in both HCs and FES patients, females outperformed males in BVMT (visuospatial memory), while males showed superiority in NAB (planning/problem-solving). Compared to HCs, FES patients exhibited global cognitive deficits, with the most pronounced impairments in CPT (sustained attention) and BACS (processing speed) across genders, while MSCEIT (emotional intelligence) showed the smallest effect sizes. Gender-dependent correlations further highlighted that cognitive impairments in male FES patients were exclusively associated with negative symptoms, specifically linked to deficits in TMT, BACS, CPT, and MSCEIT. In contrast, female FES patients demonstrated cognitive impairments (e.g., NAB) that were related to all PANSS symptom domains, underscoring distinct gender-specific relationships between cognition and psychopathology in early psychosis.

The current finding that both HC and FES patients exhibit female superiority in visuospatial memory and male advantage in planning/problem-solving aligns with neurobiological theories of sex-specific cognitive organization (31, 32). The current findings align with a study demonstrating a female advantage in jigsaw puzzle solving-a visuospatial task analogous to BVMT-where females outperformed males by leveraging distinct cognitive strategies distinct from males’ reliance on mental rotation abilities (33). Notably, a study by Sang et al. (34) reported different cortical thickness patterns related to memory in males and females among community-elderly. They found that females had thicker medial temporal and orbitofrontal cortices, which were associated with their superiority in verbal memory. In contrast, males showed enhanced lateral prefrontal and occipital thickness, linked to their advantage in visuospatial memory. This seemingly conflicts with our results of female superiority in visuospatial memory. However, the apparent contradiction might be due to the complexity of visuospatial processing. Visuospatial tasks can be divided into multiple sub-domains, such as spatial memory (as measured by BVMT in our study, focusing on retaining visual layouts) and spatial rotation/navigation. The male-related cortical thickness in lateral prefrontal and occipital regions in the Sang et al. study might underpin strengths in spatial rotation or navigation aspects, which were not evaluated in our research. Instead, our BVMT task likely engages different neural substrates, perhaps involving the medial temporal regions where females showed thickness advantages in the context of verbal memory, but which could also play a role in visuospatial memory tasks like BVMT. A study by Gogos et al. (2022) examining bipolar disorder patients found trends toward females outperforming males in visual memory (BVMT-R), though non-significant after correction, which aligns with the robust female visuospatial memory advantage observed in both HC and FES cohorts here (35).

The current finding of male superiority in reasoning and problem-solving (evident in NAB tasks) across both HC and FES groups aligns with the authors’ prior research showing that in Chinese FES patients under 35, males outperformed females in aspects of working memory and reasoning/problem-solving (3). A study by Gallagher et al. also revealed that male students demonstrated greater strategy flexibility in mathematical problem-solving, particularly in tasks requiring spatial skills or multiple solution paths (36), which aligns with the present study’s finding of male superiority in NAB (planning/problem-solving) across healthy and psychotic populations. A systematic review by Leger & Neill spanning human schizophrenia patients and rodent models corroborates the male advantage in working memory and problem-solving, while highlighting female superiority in visual memory—findings that align with the present study’s demonstration of consistent gender-specific cognitive profiles across healthy controls and first-episode schizophrenia patients (37). Notably, while our findings in drug-naïve patients align with prior research on gender-specific cognitive profiles, inconsistencies in medicated cohorts suggest that antipsychotic effects or sample heterogeneity may influence observed differences, underscoring the importance of study context for generalizability.

The current study reveals striking gender-dependent correlations between cognitive impairments and clinical symptoms in FES patients, whereby male patients exhibit cognitive deficits that are uniquely associated with negative symptoms, while female patients show broader associations between cognitive deficits and all PANSS symptom domains. The current study’s findings of sex-specific cognitive and symptom associations in FES patients are bolstered by Samizadeh et al.’s (2024) research on a rat model of schizophrenia (38), where sub-chronic ketamine administration induced sex-dependent behavioral changes paralleling the divergent cognitive-symptom relationships observed in male and female FES patients, and further emphasizing the role of sex as a critical factor in understanding psychosis-related phenotypes. The findings suggest that male FES patients may experience more circumscribed cognitive impairments linked to negative symptoms, whereas female patients show more diffuse cognitive-symptom associations, potentially reflecting sex differences in neural vulnerability or symptom expression pathways. The current study’s findings of gender-specific cognitive-symptom associations in FES patients are further contextualized by Rössler et al.’s (2015) population-based research, which showed comparable (though modest) negative associations between subclinical psychotic symptoms and processing speed in both sexes, suggesting that sex differences in psychosis-cognition links may emerge more prominently in clinical populations (e.g., FES) than at subthreshold symptom levels (39). Such dissociations highlight the need for gender-sensitive approaches in targeting cognitive and symptomatic interventions in early psychosis.

The sex-divergent cognitive profiles identified in our study carry significant clinical implications: male FES patients’ relatively preserved problem-solving ability— as shown in our findings—could be harnessed in cognitive remediation programs, as Bowie et al. (2019) (40) demonstrated that personalized training based on cognitive strengths improves long-term functional outcomes in schizophrenia. Conversely, female FES patients’ notable attention deficits (associated with negative symptoms) necessitate early attention-training interventions, such as focused exercises and cognitive-behavioral therapies, especially since Chen et al. (2022) (41) found gender-specific variations in cognitive domains among stable patients. Clinically, integrating gender-specific cognitive assessments is critical: monitoring visuospatial memory decline in females and executive function trajectories in males—supported by studies on gendered cognitive profiles in schizophrenia (41)—enables clinicians to tailor interventions for improved early psychosis treatment efficacy.

### 4.1 Limitations

The present study is subject to several limitations that warrant consideration. First, the single-center design may limit the generalizability of findings, as regional variations in healthcare access, diagnostic practices, or genetic demographics could influence results. Second, the cross-sectional nature of the research precludes causal inferences about the temporal relationship between sex-specific cognitive deficits and psychosis onset, leaving open questions about whether observed differences precede or result from the disorder. Third, the absence of IQ assessment in the cognitive battery introduces a potential confound, as baseline intellectual ability could modulate the expression of cognitive impairments in FES patients. Additionally, observed between-group differences in parental education levels may reflect socioeconomic disparities that could indirectly affect cognitive development or access to educational resources, potentially influencing performance on neuropsychological tests. Future studies employing multicenter designs, longitudinal follow-up, and comprehensive assessments of premorbid functioning (including IQ and socioeconomic status) are needed to address these limitations and further validate the sex-specific cognitive profiles identified in this study. Notably, FES patients had parents with significantly lower education levels than HCs, which may reflect socioeconomic disparities that could indirectly influence cognitive development or test performance (42). Future studies should incorporate comprehensive assessments of socioeconomic status to disentangle its impact on cognitive profiles in early psychosis.

## 5 Conclusion

This study, leveraging a large sample and rigorous methodology, has uncovered significant gender differences in cognitive performance and the relationship between cognitive impairments and psychopathological symptoms among first-episode schizophrenia patients. These findings emphasize the necessity of incorporating gender considerations in understanding, diagnosing, and treating cognitive deficits in early psychosis.

## Data Availability

The raw data supporting the conclusions of this article will be made available by the authors, without undue reservation.
